# *N*-acetylmannosamine-6-phosphate 2-epimerase uses a novel substrate-assisted mechanism to catalyze amino sugar epimerization

**DOI:** 10.1016/j.jbc.2021.101113

**Published:** 2021-08-24

**Authors:** Michael J. Currie, Lavanyaa Manjunath, Christopher R. Horne, Phillip M. Rendle, Ramaswamy Subramanian, Rosmarie Friemann, Antony J. Fairbanks, Andrew C. Muscroft-Taylor, Rachel A. North, Renwick C.J. Dobson

**Affiliations:** 1 Biomolecular Interaction Centre and School of Biological Sciences, University of Canterbury, Christchurch, New Zealand; 2 Institute of Stem Cell Science and Regenerative Medicine, NCBS, Bangalore, Karnataka, India; 3 Ferrier Research Institute, Victoria University of Wellington, Lower Hutt, New Zealand; 4 Fujirebio Diagnostics, Gothenburg, Sweden; 5 Centre for Antibiotic Resistance Research (CARe), University of Gothenburg, Gothenburg, Sweden; 6 Biomolecular Interaction Centre and School of Physical and Chemical Sciences, University of Canterbury, Christchurch, New Zealand; 7 Department of Biochemistry and Biophysics, Stockholm University, Stockholm, Sweden; 8 Department of Biochemistry and Molecular Biology, Bio21 Molecular Science and Biotechnology Institute, University of Melbourne, Parkville, Victoria, Australia

**Keywords:** epimerase, ManNAc-6P, GlcNAc-6P, sialic acid, enzyme mechanism, crystal structure, methicillin-resistant *Staphylococcus aureus*, energy metabolism, 6PG, 6-phosphogluconate, *Cp*NanE, NanE enzyme from *Clostridium perfringens*, G6P, glucose-6-phosphate, G6PD, glucose-6-phosphate dehydrogenase, GlcN-6P, glucosamine-6-phosphate, GlcNAc-6P, *N*-acetylglucosamine-6-phosphate, ManNAc-6P, *N*-acetylmannosamine-6-phosphate, NagA, GlcNAc-6P deacetylase, NagB, glucosamine-6-phosphate deaminase, NAL, *N*-acetylneuraminate lyase, NanE, *N*-acetylmannosamine-6-phosphate 2-epimerase, PDB, Protein Data Bank, PGI, phosphoglucoisomerase, *Sa*NanE, NanE from *Staphylococcus aureus*, SAXS, small-angle X-ray scattering, TIM, triosephosphate isomerase

## Abstract

There are five known general catalytic mechanisms used by enzymes to catalyze carbohydrate epimerization. The amino sugar epimerase *N*-acetylmannosamine-6-phosphate 2-epimerase (NanE) has been proposed to use a deprotonation–reprotonation mechanism, with an essential catalytic lysine required for both steps. However, the structural determinants of this mechanism are not clearly established. We characterized NanE from *Staphylococcus aureus* using a new coupled assay to monitor NanE catalysis in real time and found that it has kinetic constants comparable with other species. The crystal structure of NanE from *Staphylococcus aureus*, which comprises a triosephosphate isomerase barrel fold with an unusual dimeric architecture, was solved with both natural and modified substrates. Using these substrate-bound structures, we identified the following active-site residues lining the cleft at the C-terminal end of the β-strands: Gln11, Arg40, Lys63, Asp124, Glu180, and Arg208, which were individually substituted and assessed in relation to the mechanism. From this, we re-evaluated the central role of Glu180 in this mechanism alongside the catalytic lysine. We observed that the substrate is bound in a conformation that ideally positions the C5 hydroxyl group to be activated by Glu180 and donate a proton to the C2 carbon. Taken together, we propose that NanE uses a novel substrate-assisted proton displacement mechanism to invert the C2 stereocenter of *N*-acetylmannosamine-6-phosphate. Our data and mechanistic interpretation may be useful in the development of inhibitors of this enzyme or in enzyme engineering to produce biocatalysts capable of changing the stereochemistry of molecules that are not amenable to synthetic methods.

Enzymes catalyze chemical reactions with a high degree of stereoselectivity, an emergent property that stems from their own three-dimensional structures. Within the class of isomerase enzymes is the subclass of epimerases, which catalyze specific inversions of stereochemistry in biomolecules that have multiple chiral centers. Carbohydrates have many chiral centers, and epimerase-mediated inversions of stereochemistry yield a diverse range of structures (1). Currently, five mechanistic pathways have been proposed for carbohydrate epimerases: deprotonation–reprotonation; formation of a transient keto intermediate; carbon–carbon bond cleavage and reformation; nucleotide elimination–readdition; and mutarotation to release and then reform stereochemical centers (reviewed in Ref. (2)). Here, we propose a new epimerization mechanism involving proton displacement mediated by the substrate, drawing on structural and kinetic evidence.

Sialic acids are a diverse family of nine-carbon carbohydrates that have seven chiral centers (3). In humans, sialic acids, particularly *N*-acetylneuraminic acid, are highly abundant in the respiratory and gastrointestinal tracts (4), where they coat glycoconjugates as terminal sugars (5). Although rich in sialic acid, these environments are glucose limited, which makes bacterial survival difficult. To gain a competitive growth advantage and aid in colonization, bacteria such as *Staphylococcus aureus* overcome this nutrient limitation by scavenging, importing, and degrading host-derived sialic acid as an alternative nutrient source (Fig. 1*A*) (6). The sialic acid catabolic pathway provides a source of carbon, nitrogen, and energy and has been identified in 452 bacterial species, most of which are mammalian pathogens or commensals (7). The sequence of enzyme-catalyzed reactions in the pathway is generally well conserved across bacteria that use sialic acid, indicating that it is an essential pathway in nature (8). In addition, the mechanisms by which these enzymes function are largely understood (9, 10, 11, 12, 13, 14, 15), and the gene regulation of the pathway is currently being characterized (16, 17, 18, 19, 20). Sialic acid import and degradation has proven to be necessary for pathogen colonization and persistence *in vivo* with mouse models for *Escherichia coli* (21), *Vibrio cholerae* (22), and *Vibrio vulnificus* (4).

**Figure 1:**
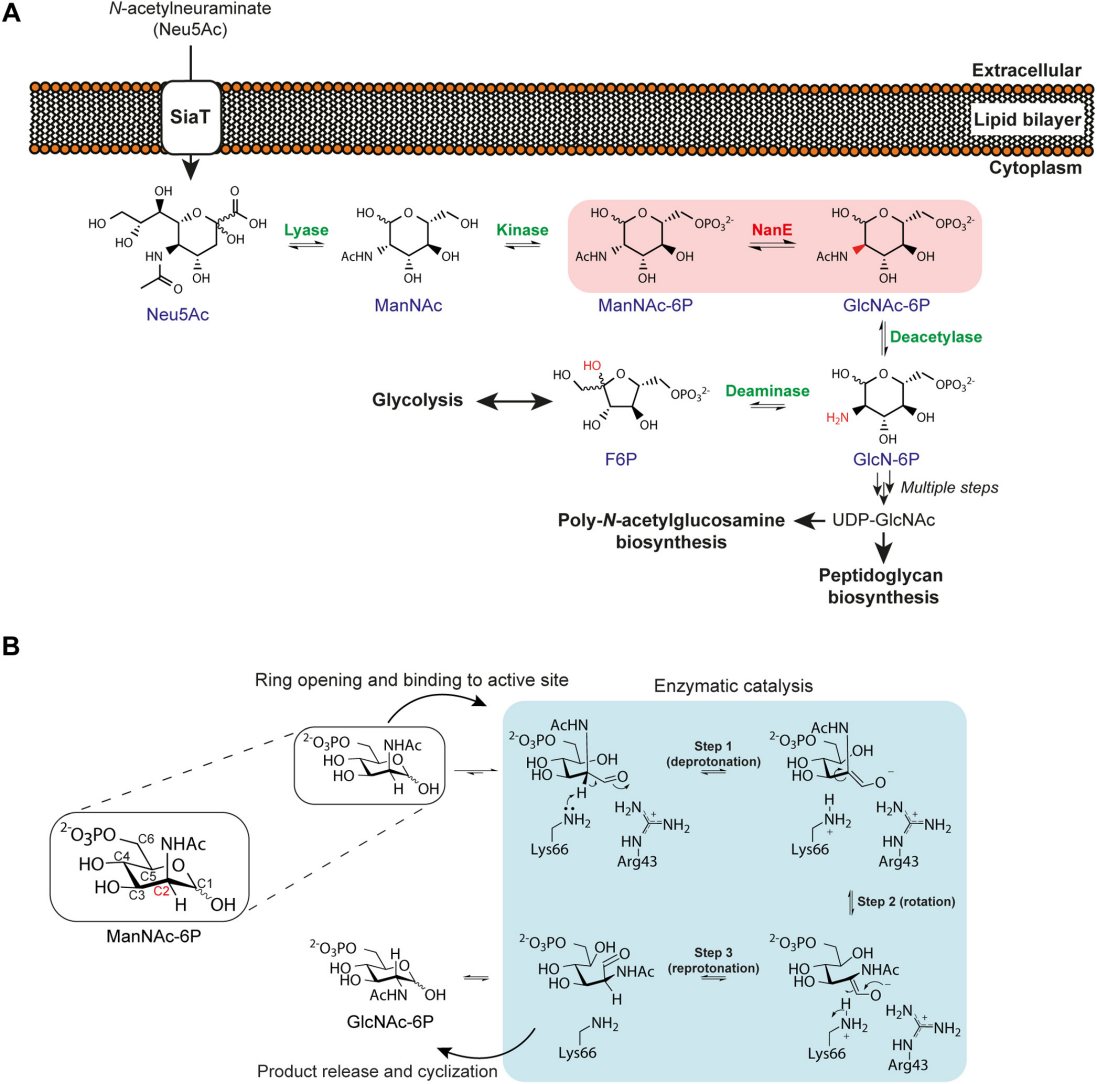
**The pathway involving NanE and its proposed mechanism.***A*, general sialic acid catabolic pathway in bacteria. The reaction of NanE (highlighted in the *red box*) inverts the stereochemistry of ManNAc-6P at the C2 position to yield GlcNAc-6P as the third step of the pathway. *B*, proposed deprotonation–reprotonation catalytic mechanism of *Cp*NanE. A proton is removed by Lys66 from an activated stereogenic center at the C2 position of ManNAc-6P (step 1). The negatively charged enolate intermediate is stabilized by Arg43. Prior to reprotonation, the enolate rotates ∼45° around the C2–C3 bond (step 2). Reprotonation by Lys66 generates the product epimer, GlcNAc-6P (step 3) (25). Residue numbering is from *Cp*NanE. *Cp*NanE, NanE enzyme from *Clostridium perfringens*; GlcNAc-6P, *N*-acetylglucosamine-6-phosphate; ManNAc-6P, *N*-acetylmannosamine-6-phosphate; NanE, *N*-acetylmannosamine-6-phosphate 2-epimerase.

The third step in the sialic acid catabolic pathway is mediated by the enzyme *N*-acetylmannosamine-6-phosphate 2-epimerase (NanE) (8), which catalyzes an inversion of stereochemistry at the C2 position of *N*-acetylmannosamine-6-phosphate (ManNAc-6P), yielding *N*-acetylglucosamine-6-phosphate (GlcNAc-6P) (Fig. 1*A*). NanE is essential for the growth of *S. aureus* in media containing *N*-acetylneuraminic acid as the sole carbon source (8) and has a highly conserved active site across both Gram-positive and Gram-negative bacteria (23). In mammals, a bifunctional UDP-*N*-acetylglucosamine 2-epimerase/*N*-acetylmannosamine kinase catalyzes the equivalent reaction in the biosynthesis of sialic acid (24). Because of its essential nature, NanE is an attractive target to inhibit pathogenic bacteria, but to guide drug development, a robust understanding of the mechanism by which NanE mediates catalysis is required.

A study by Pélissier *et al.* (25) presented the first structural and kinetic data for a NanE enzyme from *Clostridium perfringens* (*Cp*NanE), from which they derived a catalytic mechanism for inversion of the stereochemistry at the C2 position of ManNAc-6P. The authors proposed that inversion occurs *via* a deprotonation–reprotonation mechanism by a single residue, Lys66, which acts as a Brønsted base/acid (Fig. 1*B*) (25). In general, deprotonation–reprotonation mechanisms involve cleavage of a C–H bond at an “activated” stereogenic center adjacent to a carbonyl group, such as an aldehyde, ketone, or carboxylate (2, 26), which lowers the p*K*_a_ of the proton at the stereogenic center to <25. The resulting resonance-stabilized enolate facilitates proton removal (1). For ManNAc-6P, the C2 position is adjacent to an aldehyde at the C1 position, activating the stereogenic center for deprotonation. Following removal of the proton (Fig. 1*B*, step 1), the negatively charged enolate intermediate is stabilized by Arg43 (25). The authors hypothesize that the side chain of Lys66 then undergoes a minimal displacement from the substrate following (or in concert with) a repositioning of Gln14. The movement of Lys66 allows the enolate intermediate to rotate ∼45 to 50° around the C2–C3 bond (Fig. 1*B*, step 2), providing Lys66 access to the opposing face of the enolate. Reprotonation by Lys66 then generates the C2 epimer, GlcNAc-6P (Fig. 1*B*, step 3).

The work described herein is a structural and kinetic study of NanE from *S. aureus* (*Sa*NanE) to further understand the mechanism of the catalyzed reaction. By solving the crystal structure of *Sa*NanE to high resolution, with and without substrate, and, in combination with comparative sequence analysis, residues involved in catalysis were identified and tested by mutagenesis and kinetic analysis. Here, we reconsider the lysine deprotonation–reprotonation mechanism proposed by Pélissier *et al.* (25) using their submitted structures and combine it with our structural, kinetic, and modeling studies. These studies suggest that, whilst plausible, the proposed mechanism is unlikely. Based on these data, we propose an alternative mechanism for NanE catalysis—epimerization *via* a novel substrate-assisted proton displacement mechanism.

## Results and discussion

### S*a*NanE forms a stable and conserved dimer

We determined the crystal structure of *Sa*NanE to evaluate the reaction mechanism in the *S. aureus* homolog. Purified recombinant protein (see Experimental procedures section) readily produced crystals that diffracted to a maximum resolution of 1.84 Å (Table 1). The data were processed in the space group *P*2_1_2_1_2 and solved by molecular replacement (Protein Data Bank [PDB] ID: 6VVA). The active site contained an area of strong electron density, consistent with a citrate molecule at high occupancy (modeled as 0.84 by phenix.refine), which is likely derived from the crystallization conditions (Fig. S1).

**Table 1:** Data collection and refinement statistics

Parameters	Wildtype apo	Wildtype sub/prod	Wildtype 5-deoxy sub	Glu180Ala apo
Wavelength (Å)	0.95370	0.95369	0.95370	0.95366
Detector	ADSC Quantum 315r	ADSC Quantum 315r	ADSC Quantum 210r	EIGER X 16M
Space group	*P*2_1_2_1_2	*P*2_1_2_1_2	*P*2_1_2_1_2	*P*2_1_2_1_2
Cell dimensions: a, b, c (Å)	77.07, 173.74, 44.43	76.47, 174.21, 43.93	76.44, 173.45, 44.04	77.00, 174.45, 44.52
Mosaicity (°)	0.13	0.25	0.20	0.10
Resolution (Å)	46.34–1.84 (1.91–1.84)	46.25–1.51 (1.56–1.51)	46.11–1.88 (1.95–1.88)	46.40–1.55 (1.57–1.55)
Unique reflections	52,643 (5094)	91,961 (8946)	48,503 (4640)	87,951 (8254)
CC_1/2_	0.995 (0.592)	0.998 (0.661)	0.998 (0.702)	0.999 (0.842)
R_*merge*_	0.095 (0.610)	0.200 (1.235)	0.125 (1.120)	0.085 (0.675)
R_*meas*_	0.112 (0.744)	0.203 (1.279)	0.135 (1.212)	0.089 (0.705)
*I*/Σ*i*	10.8 (1.8)	17.5 (2.8)	10.1 (1.5)	13.5 (2.0)
Wilson *B*-factor	16.92	11.33	20.66	18.94
Completeness (%)	99.70 (98.87)	98.73 (97.40)	99.67 (97.13)	99.5 (89.1)
Multiplicity	3.6 (2.9)	30.1 (14.4)	7.2 (6.8)	13.1 (11.8)
Refinement				
Reflections in refinement	52,643 (5094)	91,961 (8946)	48,481 (4649)	87,951 (8245)
Reflections for R_*free*_	1993 (192)	4620 (454)	2468 (218)	4413 (415)
R_*work*_	0.1797 (0.3100)	0.1798 (0.2915)	0.1605 (0.3012)	0.1591 (0.2261)
R_*free*_	0.2005 (0.3139)	0.2099 (0.3275)	0.1981 (0.3187)	0.1817 (0.2399)
No. of nonhydrogen atoms	4296	4408	4279	4406
Protein	3683	3732	3634	3709
Ligand	28	80	64	32
Solvent	585	596	581	665
RMS deviations bonds (Å)	0.013	0.016	0.014	0.016
RMS deviations angles (°)	1.64	1.96	1.83	1.97
Average *B*-factors (Å^2^)	18.46	14.54	23.70	22.41
Macromolecules	16.43	12.21	21.71	19.97
Ligands	33.04	24.51	30.53	29.73
Solvent	30.51	27.75	35.39	35.72
Ramachandran favored (%)	96.82	96.36	97.04	96.82
Ramachandran allowed (%)	2.73	3.18	2.51	2.50
Ramachandran outliers (%)	0.45	0.45	0.46	0.68
Rotamer outliers (%)	0.24	1.65	0.98	1.20
Clash score	1.20	9.35	4.86	4.24
Protein Data Bank entry	6VVA	7MFS	7MQT	7MFN

Values in the outer shell are shown in parentheses.

**Figure 2:**
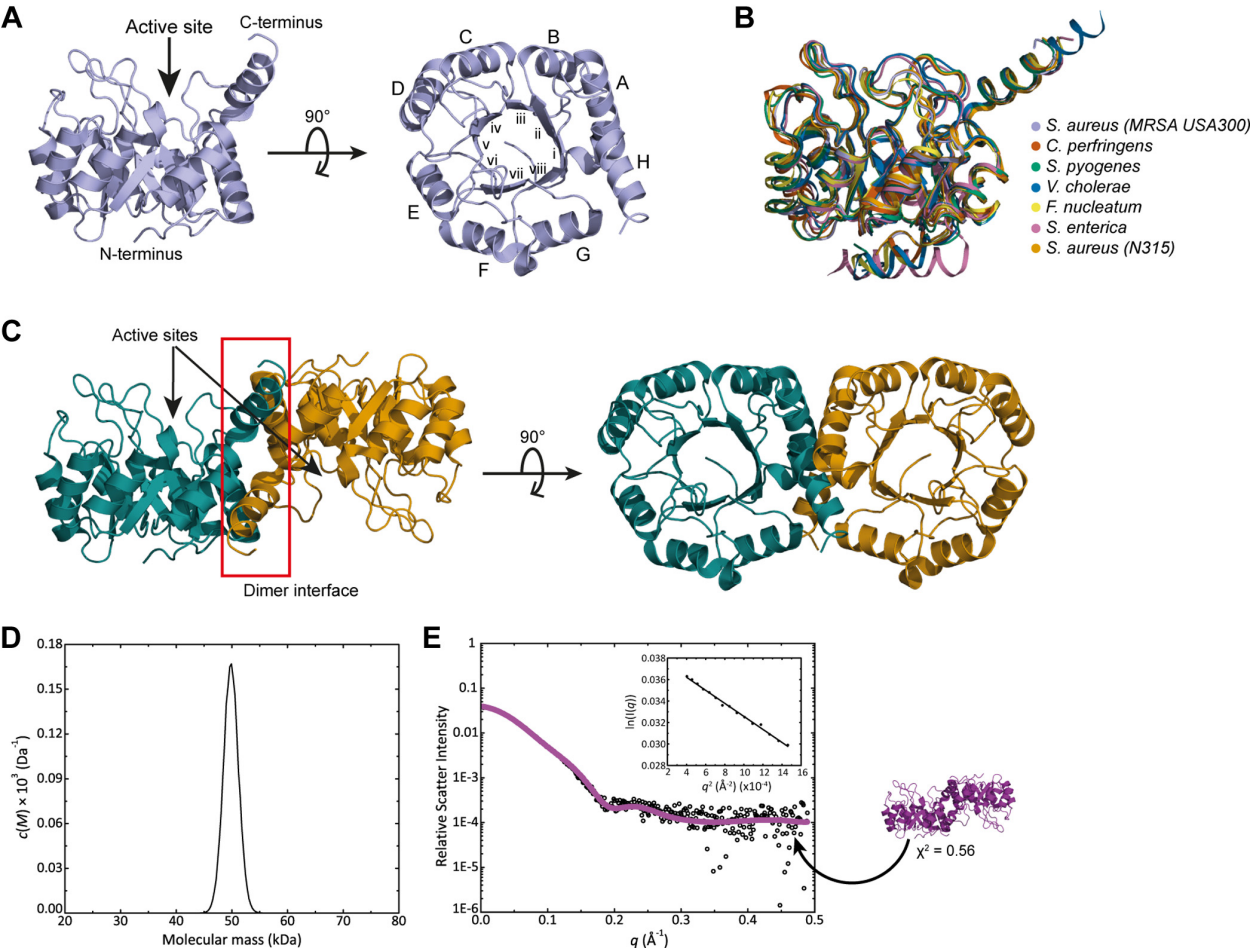
**The structure of wildtype *Sa*NanE.***A*, the monomeric structure is a TIM barrel with a unique helix at the C terminus. α-Helices (*letters*) and β-strands (*numerals*) are labeled from the N terminus to the C terminus. The active-site resides in the C-terminal loops. *B*, overlay of the *Sa*NanE monomer with orthologous NanE models available in the Protein Data Bank from *Clostridium perfringens* (RMSD = 0.379), *Streptococcus pyogenes* (RMSD = 0.572), *Vibrio cholerae* (RMSD = 0.852), *Fusobacterium nucleatum* (RMSD = 0.536), *Salmonella enterica* (RMSD = 0.834), and *Staphylococcus aureus* (strain N315) (RMSD = 0.397). *C*, dimeric crystal structure of *Sa*NanE. The crystal structure of *Sa*NanE was confirmed in solution, as determined from sedimentation velocity and small-angle X-ray scattering analysis. *D*, sedimentation velocity data for *Sa*NanE at 0.5 mg/ml was fitted to the c(M) model. A single component is evident with a mass of 49.2 kDa, consistent with the dimeric mass of 49.0 kDa that was calculated from the *Sa*NanE sequence. *E*, small-angle X-ray scattering data for *Sa*NanE were in close agreement with that of the dimeric crystal structure presented in *C*. The Guinier plot (inset) demonstrates an absence of aggregation and interparticle interference. The theoretical scattering profile of the crystal structure (overlaid in *magenta*) fits very well to the data (χ^2^ = 0.56, using CRYSOL). *Sa*NanE, NanE from *Staphylococcus aureus*; TIM, triosephosphate isomerase.

*Sa*NanE crystallized with two monomers in the asymmetric unit. The monomeric structure of *Sa*NanE is illustrated in Figure 2*A* (β-strands labeled i–viii and α-helices labeled A–H), showing that it assembles into a classic triosephosphate isomerase (TIM) barrel fold, with one exception: α-helix H does not pack against the β-barrel but instead mediates dimerization between the monomers. As with all TIM-barrel enzymes, the active site is located in the center of the barrel at the C-terminal end of the β-strands (27). Overall, the monomeric structure of *Sa*NanE is similar to other published NanE structures (Fig. 2*B*), including those of *C. perfringens* (PDB ID: 4UTT), *Streptococcus pyogenes* (PDB ID: 1YXY), *Salmonella enterica* (PDB ID: 3Q58), *Fusobacterium nucleatum* (PDB ID: 5ZKN), and *V. cholerae* (PDB ID: 5ZJB). This is demonstrated by a small variation in RMSD when each structure is compared with *Sa*NanE (ranging from 0.379 for *C. perfringens* to 0.852 for *V. cholerae*), indicating that the mechanism of catalysis is likely to be the same. It is worth noting that an *S. aureus* NanE structure is available in the PDB (PDB ID: 1Y0E). However, this was deposited by the Midwest Center for Structural Genomics and has not been reported in the literature. Our structure varies from 1Y0E by two residues, 1Y0E has an extra alanine preceding the first methionine, and Val219 in 1Y0E is substituted to Ile219.

Unlike other TIM-barrel enzymes, dimerization between the *Sa*NanE monomers is enabled by a C-terminal α-helix, which projects away from each barrel and packs against the neighboring monomer (Fig. 2*C*). Using analytical ultracentrifugation and small-angle X-ray scattering (SAXS), we verified that the solution structure and shape is consistent with our crystal structure. Sedimentation velocity experiments demonstrated that wildtype *Sa*NanE is a homodimer in solution. The continuous sedimentation distribution model resulted in a single symmetrical peak, indicating that wildtype *Sa*NanE is a single species in solution, with a sedimentation coefficient of 3.5 S (Fig. S2*A* and *B*). When the data were fitted to a continuous mass distribution model, the apparent molecular mass was 49.2 kDa (Fig. 2*D*), consistent with the dimeric mass of 49 kDa calculated from the *Sa*NanE amino acid sequence. SAXS data further verified the dimeric structure of *Sa*NanE in solution. Scattering data are presented as an intensity plot (Fig. 2*E*). The linearity of the Guinier plot confirmed the absence of aggregation and interparticle interference (Fig. 2*E*, inset). The maximum dimension of the scattering particle (*D*_max_) was 75.8 Å (Table 2), as calculated using AUTOPOROD (28). A radius of gyration of 24.9 Å was determined by GNOM (29) and was analogous to that determined by Guinier analysis (23.5 Å). The molecular mass extrapolated from the SAXS data was 40.4 kDa, which although smaller than the 49 kDa theoretical dimeric mass and the apparent molecular mass derived from sedimentation velocity analysis (49.2 kDa), it is most consistent with a dimeric structure. To determine whether the solution structure accurately reflects the wildtype crystal structure, the experimental scattering data were compared with a theoretical scattering profile generated from the crystal structure using CRYSOL (30). The theoretical scattering profile calculated for the wildtype crystal structure of *Sa*NanE was a good fit for the experimental scattering data (χ^2^ = 0.56), suggesting that the crystal structure is an accurate representation of the solution structure (Fig. 2*C*). The *P*(*r*) distribution (Fig. S2*C*) showed a single peak with a positively skewed tail at long distances, indicating that *Sa*NanE has an elongated shape in solution, consistent with the crystal structure of the dimer.

**Table 2:** SAXS data collection and analysis statistics

SAXS data analysis	*Sa*NanE
*R*_g_ (Å) (from Guinier analysis)	23.5
*R*_g_ (Å) (from *P*(*r*) analysis)	24.9
*D*_max_ (Å)	75.8
Molar mass (from Porod volume, kDa)	40.4
Calculated dimer mass from sequence (kDa)	49.0
SAXS data-collection parameters	
Instrument	Australian Synchrotron SAXS/WAXS beamline
Detector	PILATUS 1M (Dectris)
Wavelength (Å)	1.0332
Maximum flux at sample	8 × 10^12^ photons per second at 12 keV
Camera length (mm)	1600
*q* range (Å^−1^)	0.006–0.4
Exposure time	Continuous 2-s frame measurements
Sample configuration	SEC-SAXS
Sample temperature (°C)	20

### Analysis of the active site

**Figure 3:**
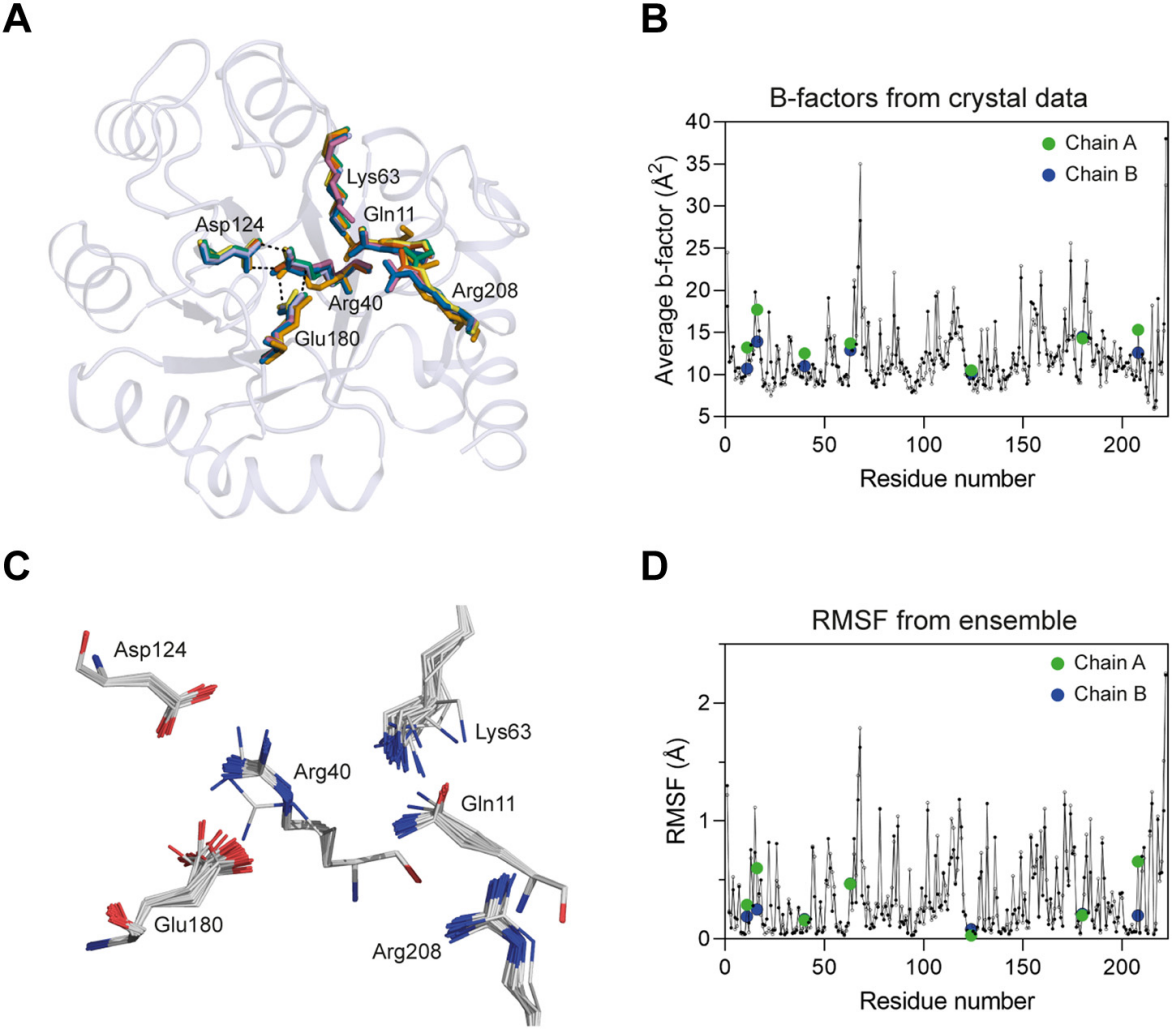
**The active site of NanE.***A*, overlaid active-site residues show the conservation of residue positions between species. *B*, temperature factor analysis of *Sa*NanE. Chain A (*solid circles*) and chain B (*open circles*) are plotted. Active-site residues are highlighted in *green* in chain A and *blue* in chain B. *C*, ensemble refinement analysis of the active-site residues of *Sa*NanE from an ensemble of 43 models. Limited variability in the residue positions was observed from our crystal data. *D*, root-mean-square fluctuation (RMSF) analysis of *Sa*NanE plotted as in *B*. NanE, *N*-acetylmannosamine-6-phosphate 2-epimerase.

We overlaid the *Sa*NanE active site with those of *C. perfringens* (PDB ID: 4UTT), *S. pyogenes* (PDB ID: 1YXY), *S. enterica* (PDB ID: 3Q58), *F. nucleatum* (PDB ID: 5ZKN), and *V. cholerae* (PDB ID: 5ZJB) and determined that the key active-site residues are conserved and positioned with near identity in all structures (Fig. 3*A*). Using the *C. perfringens* structure with bound ManNAc-6P, the key active-site residues lining the cleft at the C-terminal ends of the β-strands in *Sa*NanE were identified as Gln11, Arg40, Lys63, Asp124, Glu180, and Arg208. A broader sequence alignment with 11 bacterial NanE enzymes further demonstrated that the active-site residues are highly conserved (Fig. S3). Moreover, two signature sequence motifs comprising X-Pro-X-Ile-Gly-Ile-X-Lys-Z and Val-Gly-U-Ala-X-Thr-Arg, where X is a hydrophobic residue, Z is a basic residue, and U is Ser or Gly, are present in all the compared sequences and may be useful for identifying further bacterial NanE enzymes.

We examined temperature factors for the residues on each monomer to gauge whether the active-site residues are mobile, as suggested in the mechanism proposed by Pélissier *et al.* (25) (the side chains of Lys66 and Gln14 [Lys63 and Gln11 in *Sa*NanE] move to allow the substrate to rotate). In general, the active-site residues map to regions of the protein that have low temperature factors, as evidenced by an overlay of active-site residues on the temperature factor plot for each chain in the asymmetric unit (Fig. 3*B*). To further gauge the dynamics of the active-site residues, we conducted an ensemble refinement of the structure and again found that the active-site residues have low root-mean-square fluctuations and largely occupy a single conformation (Fig. 3, *C* and *D*). In the context of the mechanism, Pélissier *et al.* (25) modeled Gln14 in two alternative conformations, and this is taken as evidence that the mobility of Gln14 facilitates the conformational change in Lys66 to allow the substrate to rotate. Here, we find that Gln11 does not adopt alternative conformations in *Sa*NanE or in structures solved from other bacterial species.

The estimated p*K*_a_ values of the proposed catalytic residues were calculated using PROPKA 3.0 (31, 32, 33) to assess their likely role in catalysis. In the apo structure, two active-site residues have side-chain moieties with significantly perturbed p*K*_a_ values: Lys63 has a predicted p*K*_a_ of 8.0, considerably lower than the p*K*_a_ for lysine in water (10.4), whereas Glu180 has a predicted p*K*_a_ of 7.1, which is considerably higher than the p*K*_a_ for glutamic acid in water (4.5). The calculated p*K*_a_ for Glu180 of 7.1 suggests that the carboxylate will be protonated more than expected at the pH where the kinetic assays were performed (pH 8) (described later). The carboxylate group of Glu180 points toward the plane of the guanidinium group (3.2 Å) of the conserved Arg40, which may explain the perturbed p*K*_a_. Similarly, with Lys63, a proportion of the free amine would be present at pH 8.

Overall, we find that the structure of *Sa*NanE is very similar to those of homologous proteins studied to date. Our results suggest that the active site is rigid, although this may be a consequence of the apo form. Finally, we find that two active-site residues have significantly perturbed p*K*_a_ values: Lys63 and Glu180.

### Crystal structure of *Sa*NanE with ManNAc-6P suggests an alternative catalytic mechanism

We solved the structure of *Sa*NanE bound to its substrate, ManNAc-6P, to 1.51 Å resolution, allowing for a detailed examination of the active site and the bound substrate conformation (PDB ID: 7MFS) (Table 1).

The overall structure of the substrate-bound enzyme is highly similar to the apo structure (dimer superposition RMSD = 0.320 for 440 α-carbon atoms; monomer superposition RMSD = 0.128 for 183 α-carbon atoms [chain A], RMSD = 0.174 for 195 α-carbon atoms [chain B]). The positions of the active-site residues are unchanged—the RMSD for the overlay of the active-site residues (Gln11, Arg40, Lys63, Asp124, Glu180, and Arg208) is 0.188.

**Figure 4:**
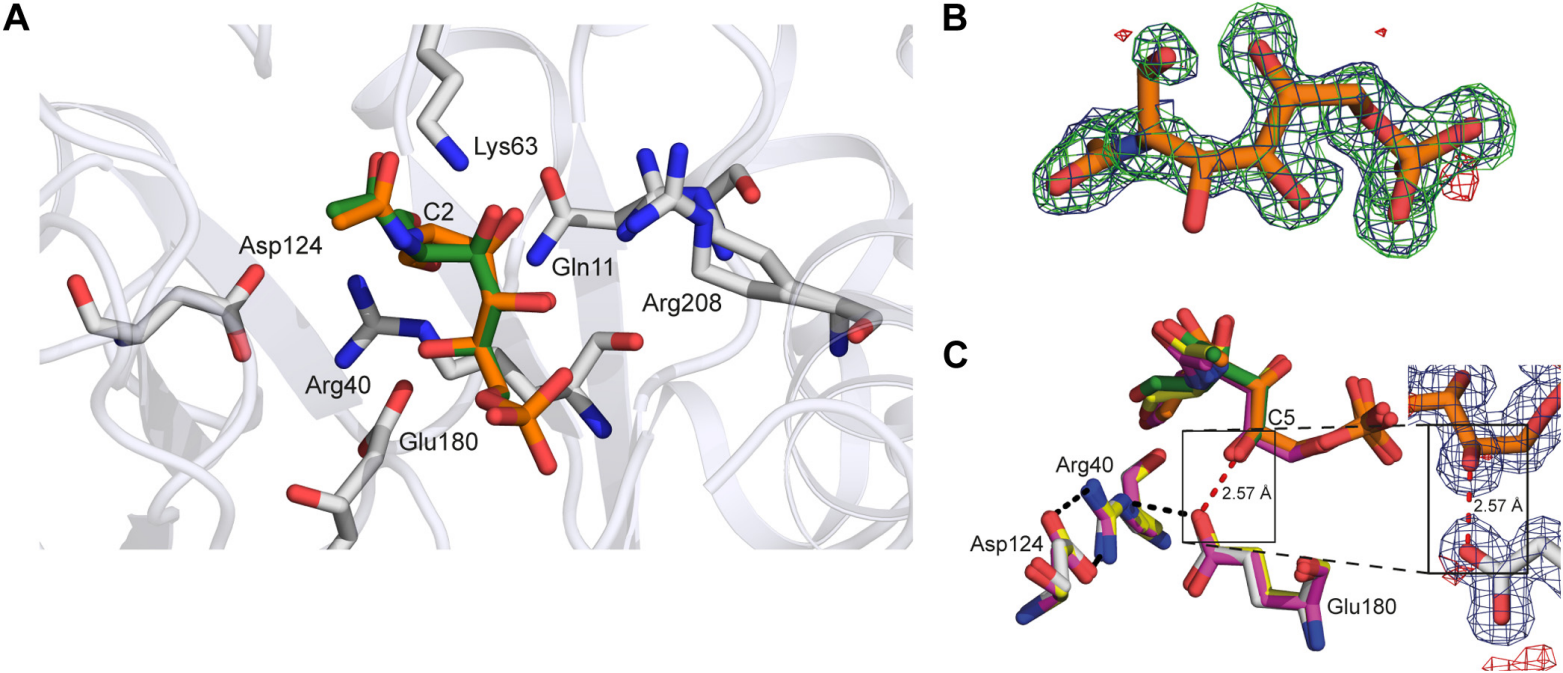
**Structure of the substrate-bound enzyme.***A*, the positioning of the open chain forms of both ManNAc-6P (*orange*) and GlcNAc-6P (*green*) in the active site of *Sa*NanE. *B*, an omit map of the active-site density (ManNAc-6P overlaid) shows clear density for the phosphorylated sugar. The 2*F*_o_–*F*_c_ electron density map is contoured at 1.0 RMS (*blue mesh*), and the *F*_o_–*F*_c_ omit electron density map is contoured at +3.0 σ (*green mesh*) and −3.0 σ (*red mesh*). *C*, the hydrogen-bond length between Glu180 and substrate/product in *Sa*NanE and *Cp*NanE is unusually short (*red bond* shown for *Sa*NanE Glu180 to ManNAc-6P, 2.57 Å). *Cp*NanE structures and ligands are colored *purple* (Protein Data Bank ID: 4UTU) and *yellow* (Protein Data Bank ID: 4UTW). The density for the oxygen atoms involved in the short bond is well defined, providing confidence that the bond is not because of ambiguous modeling. The 2*F*_o_–*F*_c_ electron density map is contoured at 1.0 RMS (*blue mesh*), and the *F*_o_–*F*_c_ omit electron density map is contoured at +3.0 σ (*green mesh*) and −3.0 σ (*red mesh*). *Cp*NanE, NanE enzyme from *Clostridium perfringens*; GlcNAc-6P, *N*-acetylglucosamine-6-phosphate; ManNAc-6P, *N*-acetylmannosamine-6-phosphate; *Sa*NanE, NanE from *Staphylococcus aureus*.

*Sa*NanE engages the ligand in an open chain form (Fig. 4*A*). As shown in an omit map, electron density corresponding to the ligand was unambiguously observed for the phosphate, *N*-acetyl and hydroxyl groups (Fig. 4*B*). Because the enzyme is catalytically functional, the active site is predicted to include both the substrate, ManNAc-6P, and the product, GlcNAc-6P, which differ in stereochemistry at the C2 position. We modeled the occupancy of the ligands with phenix.refine (34), which suggested that the active site contained 48% substrate and 52% product, in line with a previously determined equilibrium of 43% to 57% substrate to product for *E. coli* K1 NanE (35).

Overall, the protein–ligand interactions of the active site of *Sa*NanE are highly similar to those reported for *Cp*NanE (PDB ID: 4UTW), the only other ligand-bound homolog with a high-resolution structure. The catalytic lysine residue, Lys63, is completely extended toward the C2 position of the substrate/product, and the stereochemistry of C2 of ManNAc-6P positions the hydrogen toward the ε-amino group of Lys63.

Curiously, electron density was observed linking Lys63 and the C2 carbon (Fig. S4). Fitting a Lys63-C1 protein–substrate imine into this density provided a potential explanation. This led us to test whether the catalytic mechanism involves the formation of an imine. Two approaches were used to determine whether *Sa*NanE forms an imine, as is observed for its upstream catabolic partner, *N*-acetylneuraminate lyase (NAL), but which is unlike any other characterized carbohydrate epimerase. Spectrophotometric analysis (described later) of borohydride-treated *Sa*NanE revealed a 3% decrease in the reaction rate relative to untreated enzyme, whereas the activity of NAL from methicillin-resistant *S. aureus* was completely abolished. These results agreed with mass spectrometry analysis. No change in mass was observed for the treated *Sa*NanE enzyme; however, an increase in mass of ∼75 Da was noted for the treated NAL enzyme, corresponding to the formation of an imine between the enzyme and pyruvate. Both mass spectrometry and kinetic analyses indicated that an imine is not an intermediate in the mechanism of *Sa*NanE. The observed electron density may be a result of capturing proton transfer between Lys63 and C2 of the substrate, with the two groups positioned close together (2.6 Å).

The ligands (substrate and product) are anchored by hydrogen-bond formation between the phosphate group and the backbone nitrogen atoms of Gly181, Asn182, Gly203, and Gly204. We observed an extensive interaction network with water molecules, five of which interact with the phosphate group at 2.8 Å or less. The ManNAc-6P/GlcNAc-6P hydroxyl groups interact with residues Lys63 (C3), Arg208 (C3 and C4), and Glu180 (C5). Gln11, Lys63, and Arg40 are positioned in a triangle around the C1 aldehyde. The close proximity of these three residues suggests they may be involved in stabilizing the C1 oxyanion that forms in the proposed enolate intermediate. The acetamide carbonyl interacts with Lys63, and the acetamide methyl group interacts with the aromatic ring of Tyr149. These interactions are all present in both the substrate and product and in the *Cp*NanE structure. The only observable difference between the active sites of *Sa*NanE and *Cp*NanE was the presence of Phe72 in *Sa*NanE in place of Tyr75, resulting in a loss of coordination between the tyrosine hydroxyl and the carbonyl oxygen of the *N*-acetyl group.

A notable observation is the presence of a short hydrogen bond between the carboxylic acid group of Glu180 and the C5 hydroxyl of the substrate (Fig. 4*C*). Hydrogen bonding generally occurs at distances between 2.8 and 3.5 Å. However, this unusually short bond is present in our structure at an average distance of 2.6 Å (Table S1), resulting in a strong hydrogen-bond character (36, 37). This unusually short hydrogen bond is also present in the high-resolution structures from *Cp*NanE (PDB IDs: 4UTU and 4UTW), with an average bond length of 2.54 Å (Table S1), suggesting a conserved binding orientation. The lower resolution structures for *Vc*NanE appear to have a longer hydrogen bond length, although this is likely because of their lower resolution (structures of 2.66 Å) and the comparatively poor density around the ligand.

The predicted p*K*_a_ from PROPKA for Lys63 decreases from 8.0 to 5.1 upon substrate binding, reflecting the interaction with the C2 carbon. Similarly, the p*K*_a_ for Glu180 increases from 7.1 to 7.9, reflecting the short hydrogen-bonding interaction with the C5 hydroxyl.

### Kinetic characterization of S*a*NanE

**Figure 5:**
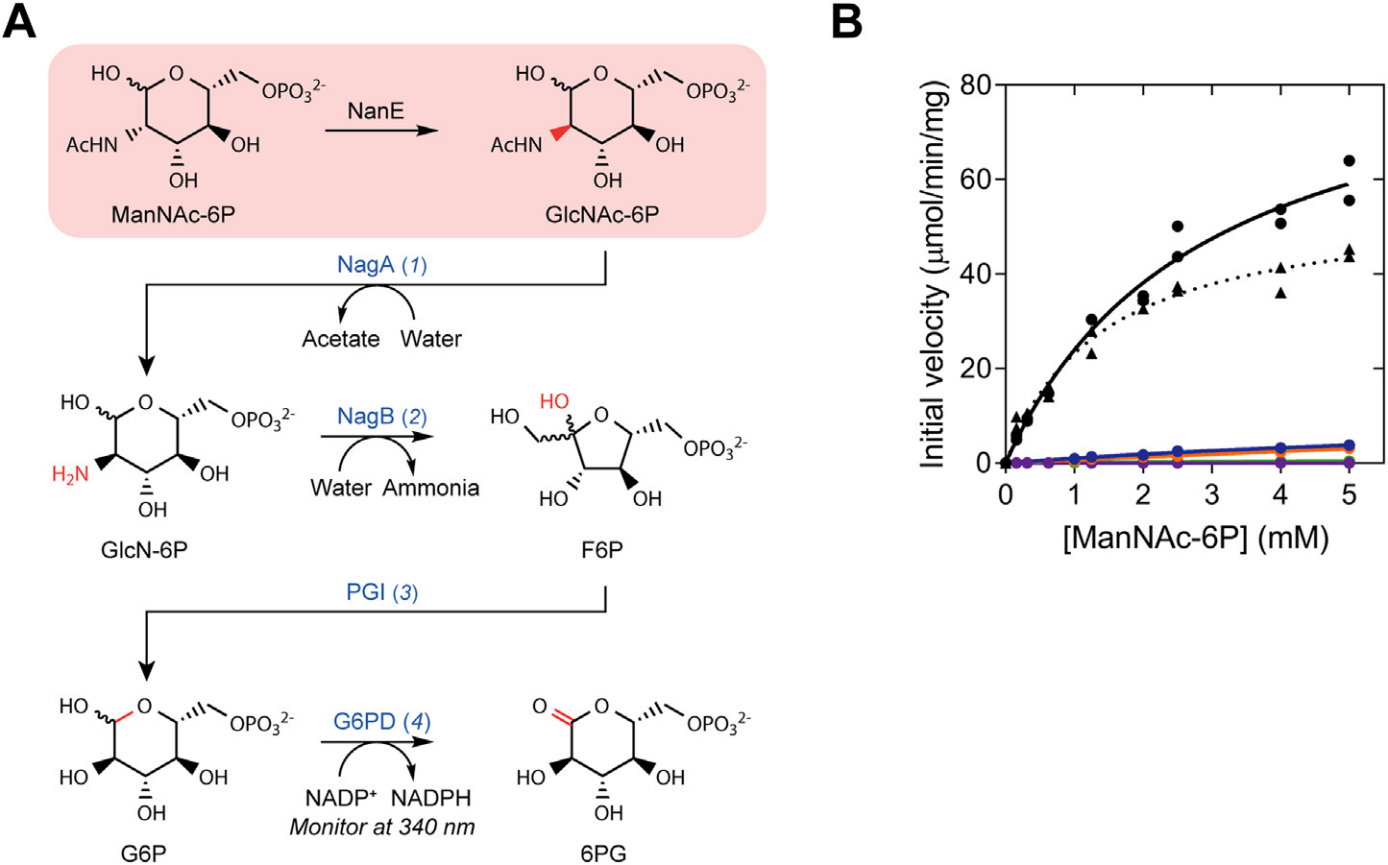
**Multienzyme coupled assay developed for NanE.***A*, as NADP^+^ is reduced to NADPH, the increase in absorbance at 340 nm is determined spectrophotometrically and is proportional to the activity of NanE. Coupling enzymes are labeled in *blue*. *B*, Michaelis–Menten kinetic analysis of *Sa*NanE enzymes. Initial velocity was plotted as a function of substrate concentration. Kinetics of the wildtype enzyme from the spectrophotometric assay (*black circles*), the wildtype enzyme from the ^1^H NMR assay (*black triangles*), and the substituted enzymes from the spectrophotometric assay Gln11Ala (*blue*), Gln11Ser (*orange*), Glu180Ala (*purple*), and Arg208Ala (*green*). NanE, *N*-acetylmannosamine-6-phosphate 2-epimerase; *Sa*NanE, NanE from *Staphylococcus aureus*.

We determined the functional properties of the enzyme by analyzing kinetic data obtained using a real-time continuous spectrophotometric coupled assay that we developed (Fig. 5*A*) (38). We compared this assay to the existing NMR-based assay, which is limited by a several minute delay prior to initial rate measurement. The spectrophotometric assay couples the reaction of interest with subsequent enzymes from the sialic acid degradation pathway, namely GlcNAc-6P deacetylase (NagA) and glucosamine-6-phosphate (GlcN-6P) deaminase (NagB), as well as phosphoglucoisomerase (PGI) and glucose-6-phosphate (G6P) dehydrogenase (G6PD) from outside the pathway. The assay is initiated by the addition of ManNAc-6P, which is converted into GlcNAc-6P by NanE. GlcNAc-6P is then sequentially converted to GlcN-6P, fructose-6-phosphate, G6P, and 6-phosphogluconate (6PG) by NagA, NagB, PGI, and G6PD, respectively. In the final step, G6PD converts G6P into 6PG, which is coupled to NADP+ reduction to NADPH (enabling measurement of NADPH absorbance at 340 nm). In the assay, the rate of this reaction is proportional to the rate of catalysis by NanE. We have previously used this assay to determine the kinetic parameters of the NanE enzymes from *F. nucleatum* and *V. cholerae* (38). A more detailed report of the assay is provided in the Supporting Information and Fig. S5.

To validate the assay, we determined the kinetic properties of wildtype *Sa*NanE and compared these results with those obtained using the established NMR assay. The initial rate data obtained for *Sa*NanE using both methods fitted well to the Michaelis–Menten model (Fig. 5*B*) and gave similar kinetic parameters (*V*_max_, *k*_cat_, and *K*_M_) (Table 3). The *K*_M_ for wildtype *Sa*NanE is in the low millimolar range, consistent with the other assayed NanE enzymes from *C. perfringens* (25), *F. nucleatum*, and *V. cholerae* (38). The *k*_cat_ values are also similar for *Sa*NanE (38 ± 3 s^−1^, spectrophotometric assay, 23 ± 1 s^−1^, NMR assay) and the NanE enzymes from *F. nucleatum* (∼41 s^−1^ (38)) and *C. perfringens* (∼170 s^−1^ (25)). This kinetic assay will be of use to others in the field for characterizing NanE enzymes and/or designing inhibitors to these enzymes, offering an alternative to NMR.

**Table 3:** Kinetic parameters of wildtype and substituted variants of *Sa*NanE

Enzyme	*V*_max_ (μmol/min/mg)	*k*_cat_ (s^−1^)	Relative *k*_cat_ (%)	*K*_*M*_ (mM)	*k*_cat_/*K*_*M*_ (s^−1^/mM)
Wildtype (our assay)	94 ± 7	38 ± 3	100	2.9 ± 0.5	13
Wildtype (NMR assay)	55 ± 3	23 ± 1	-	1.4 ± 0.2	16
*Sa*NanE-Lys63Ala	ND	ND	—	ND	—
*Sa*NanE-Lys63Glu	ND	ND	—	ND	—
*Sa*NanE-Glu180Ala	0.047 ± 0.004	0.019 ± 0.002	0.05	4.4 ± 0.6	0.004
*Sa*NanE-Arg40Ala	ND	ND	—	ND	—
*Sa*NanE-Asp124Ala	ND	ND	—	ND	—
*Sa*NanE-Asp124Gln	ND	ND	—	ND	—
*Sa*NanE-Gln11Ala	12 ± 2	4.7 ± 0.9	12	10 ± 3	0.46
*Sa*NanE-Gln11Ser	33 ± 20	14 ± 8	35	50 ± 33	0.27
*Sa*NanE-Arg208Ala	3.4 ± 1.8	1.4 ± 0.7	3.7	31 ± 19	0.04

Abbreviation: ND, not detectable.

*V*_max_, *k*_cat_, and *K*_*M*_ values (±standard error of the mean) were calculated using OriginPro or GraphPad Prism. Relative *k*_cat_ values were determined with respect to the spectrophotometric assay.

### Site-specific substitutions of active-site residues suggest catalytic roles

Pélissier *et al.* (25) proposed that Lys66 of *Cp*NanE plays a central role as a Brønsted base and acid, accepting the proton from the C2 carbon of the substrate and then donating a proton to the opposing face of the C2 carbon. Our structure of *Sa*NanE shows that the side chain amine of the equivalent residue Lys63 is well positioned to remove a proton from the C2 of the substrate (Fig. 4). Consistent with this residue playing a key role in catalysis, the substitution of Lys63 to Ala63 or Glu63 produced enzymes with no detectable activity, even at enzyme concentrations 1000-fold greater than those of wildtype *Sa*NanE (Table 3).

The active site of all NanE enzymes solved to date have a salt bridge formed between Arg40 and Asp124. The guanidinium group of Arg40 is close to the aldehyde group of the substrate (3.0 Å), and it forms a salt bridge with Glu180. To test the importance of these interactions, we generated *Sa*NanE-Arg40Ala, *Sa*NanE-Asp124Ala, and *Sa*NanE-Asp124Gln. Each of these substitutions abolished activity (Table 3), suggesting that Arg40 and Asp124 play key roles in catalysis, perhaps by altering the p*K*_a_ of Glu180, such that it can form the observed short hydrogen bond or by stabilizing the C1 oxyanion intermediate that forms during catalysis. Pélissier *et al.* (25) also reported that the substitution of the equivalent residues of Arg40, Lys63, and Asp124 in *Cp*NanE abolished enzyme activity (25).

We observed in the substrate-bound structure that Gln11 and Arg208 directly interact with the substrate. To verify their roles in substrate binding, we generated the substituted enzymes *Sa*NanE-Gln11Ala, *Sa*NanE-Gln11Ser, and *Sa*NanE-Arg208Ala. Each of these substituted enzymes retained some activity (13%, 35%, and 4%, respectively) relative to the wildtype enzyme (Table 3). However, the key functional change in these enzymes was an increase in the *K*_*M*_ (from 2.9 mM to 10, 50, and 31 mM, respectively), consistent with their proposed role in binding the substrate.

As Glu180 forms a short hydrogen bond with the C5 hydroxyl of the substrate, we generated *Sa*NanE-Glu180Ala and tested the effect of this substitution on activity. We found that *Sa*NanE-Glu180Ala retained ∼0.05% activity, but the *K*_*M*_ was similar to that of the wildtype enzyme (Table 3), suggesting that Glu180 plays a role in the catalytic mechanism. The residual activity of the enzyme may result from an active-site water molecule that acts in place of Glu180 during catalysis, a phenomenon we have seen before in another unrelated TIM-barrel enzyme, dihydrodipicolinate synthase (39).

To identify the active-site changes that led to a 2000-fold reduction in *k*_cat_ of the Glu180Ala substitution relative to the wildtype enzyme, we solved the structure of *Sa*NanE-Glu180Ala (PDB ID: 7MFN) to a resolution of 1.55 Å (Table 1). The single-residue substitution made no change to the overall structure or the conserved active-site residues. The salt bridge between Arg40 and Asp124 was preserved to stabilize the enolate, and the *K*_*M*_ was similar to wildtype *Sa*NanE (Table 3), indicating that the short bond to the C5 hydroxyl makes only a minor contribution to substrate binding. This suggests that the reduction in *k*_cat_ is due to the loss of Glu180 in the reaction mechanism rather than a compromise in substrate binding. A significant (∼50-fold) reduction in *k*_cat_ was also seen in a *Cp*NanE Glu180 substitution (25).

The lack of activity within this series of substituted enzymes could be attributed to a destabilization of the enzymes. To test this, differential scanning fluorimetry was performed to measure the thermal stability of each substituted enzyme (Table S2). Wildtype NanE has a melting temperature of 40 °C, and this was retained in the substituted enzymes, suggesting that they are not destabilized.

### Modification of the natural substrate, ManNAc-6P

We aimed to examine the involvement of the C5 hydroxyl of ManNAc-6P in the catalytic mechanism. Two derivatives were synthesized with substitutions at the C5 hydroxyl: 5-deoxy-ManNAc-6P, with a substituted hydrogen, and 5-methyl-ManNAc-6P, with a substituted methoxy group (40). *Sa*NanE was cocrystallized with these ligands to obtain ligand-bound structures.

For 5-methyl-ManNAc-6P, citrate density did not allow for ligand fitting. The increased size of the methoxy group and the proximity to Glu180 may have reduced the affinity for binding.

For 5-deoxy-ManNAc-6P, a structure was obtained to 1.88 Å resolution with density for the ligand (PDB ID: 7MQT) (Table 1). Unambiguous density for the *N*-acetyl and phosphate groups of the ligand was present in one of the monomers in the same position as in ManNAc-6P (Fig. S6). The ligand was fit to an occupancy of 0.66 with phenix.refine (34), with citrate fitted to an occupancy of 0.34. The center of the ligand appears to bind in a different orientation to that of ManNAc-6P, notably with Glu180 interacting with the C4 hydroxyl of the substituted ligand (Fig. S7). With the ability to form a hydrogen bond at the C5 position removed in 5-deoxy-ManNAc-6P, Glu180 has formed a short hydrogen bond (2.5 Å) with a different hydroxyl group.

### Modeling the previously proposed mechanism

We modeled the lysine deprotonation–reprotonation mechanism proposed by Pélissier *et al.* (25) and suggested that it is unlikely given the following reasoning:

1. A 45° rotation of the C2–C3 bond of the intermediate (Fig. 1*B*, step 2) does not provide access to the opposite face for reprotonation. We modeled this by rotating the bond up to 50°, which demonstrates that the ε-amino nitrogen remains below the plane of the enolate by 20° (Fig. 6*A*, *orange line*) and does not position the Lys66 proton for epimerization. In the product, the proton would be positioned 109° above the plane, far away from its position below the plane. Moreover, across all our structures, we see a rigid active site, which is counterintuitive for accommodating a 45° rotation of one end of the substrate.

**Figure 6:**
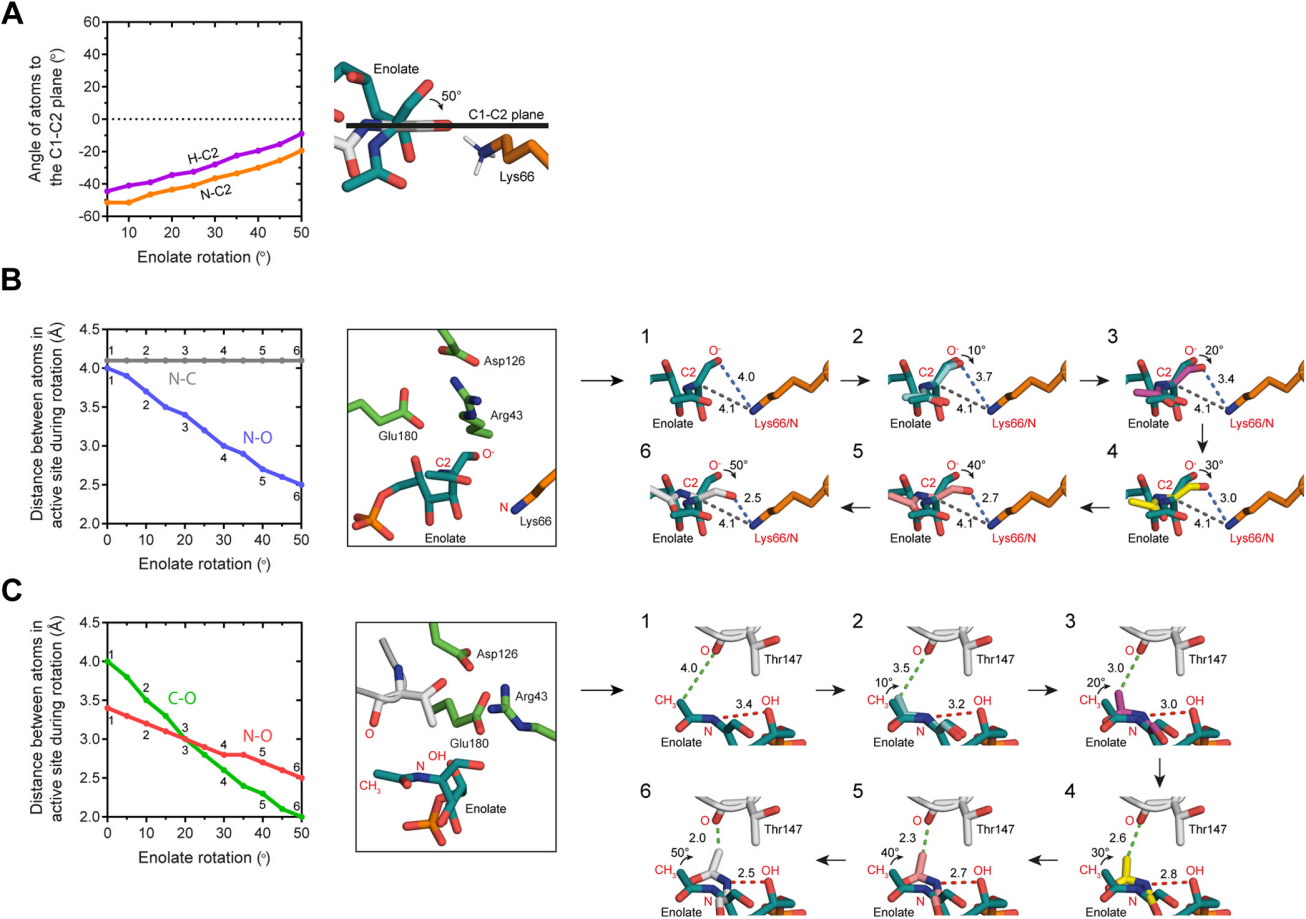
**Rotation of the intermediate.***A*, holding C3–C6 constant and rotating the C2–C3 bond causes movement of the C1–O1 and C1–C2 plane. At 40 to 50°, this rotation is insufficient to present Lys66 to the si face to enable epimerization. *B*, rotation of the C2–C3 bond would bind the enolate oxyanion in closer proximity to the NH_3_^+^ of Lys66 (4–2.5 Å), potentially facilitating rotation, but there will be energy required to disengage on product release. At the same time, the C2 stereocenter moves no closer to Lys66. Distances were measured after Lys66 was moved away to allow for rotation. *C*, steric clashes arise between Thr147 and the enolate during rotation.
2a. If the enolate does rotate, then the C1 oxyanion would come into close proximity with the ε-amino group of Lys66 (as close as 2.6 Å [Fig. 6*B*, *blue line*]), which would presumably allow for reprotonation at the oxyanion to form a hydroxyl group at C1.
2b. Even with minimal movement of Lys66 to make room for enolate rotation, the distance between the ε-amino group and C2, which is to be protonated, moves from approximately 3 to 4 Å, and is maintained during enolate rotation, making reprotonation less likely (Fig. 6*B*, *gray line*).
3. Rotation around the C2–C3 bond by 45° requires a series of protein–substrate hydrogen bonds to be broken and the displacement of an ordered (and conserved) water molecule (example in PDB ID: 4UTW, chain C, water 2169). Furthermore, electrostatic interactions between the C1 oxyanion and Arg43 and Gln14 are extended from 3.2 to 4.6 Å and 2.8 to 3.8 Å, respectively. Because the deprotonated enolate intermediate is positioned similarly to that of the substrate, the bonding network should remain intact. Therefore, how the rotation is initiated is unexplained. There is no evidence in structures that have been solved to date for such a conformational change in the substrate or protein; all reported structures have a highly conserved active site, with the residue corresponding to Lys66 in the same conformation in all structures (*i.e.*, not moved). In addition, the substrate/product is in a conserved conformation across structures, with no suggestion of rotation.
4. Modeling of the enolate demonstrates that rotation is limited to approximately 50°, as steric clashes arise between the enolate and the active site (Fig. 6*C*). In the reverse mechanism, the product (GlcNAc-6P) must bind, rotate, and displace Lys66, become deprotonated at the C2 position, form the enolate, and then rotate back to the original binding position to be reprotonated. This leads to further questions about how the rotation is initiated, as the unmodified product would have to rotate to begin catalysis or bind in an already rotated position. Even with these concerns, the mechanism presented by Pélissier *et al.* (25) could still be functional, but it seems implausible given the data presented, as others have noted (41).

### A new mechanism for NanE catalysis

We observed a rigid active site with a strong bonding network to the substrate ManNAc-6P that positions the substrate/product in a specific and conserved pseudochair conformation. Analysis of B-factors indicates low movement within the active-site residues. Mutagenesis studies identified the roles of Lys63 and Glu180, and crystallographic modeling highlighted the unusually short hydrogen bond between Glu180 and the substrate, which is retained even if the C5 hydroxyl of the substrate is removed (it now forms with the C4 hydroxyl).

**Figure 7:**
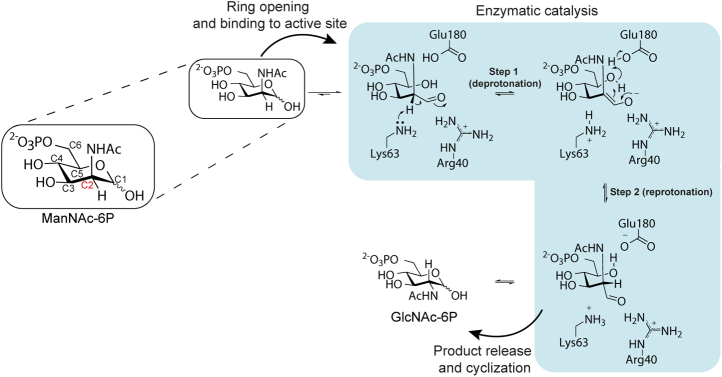
**Proposed proton displacement mechanism of *Sa*NanE.** Lys63 removes the proton at the activated C2 position of ManNAc-6P (step 1). The enolate intermediate formed by deprotonation is stabilized by Arg40. The substrate hydroxyl at the C5 position is activated by Glu180 and reprotonates the C2 position on the opposite face to generate the product epimer, GlcNAc-6P (step 2). Residue numbering is from *Sa*NanE. GlcNAc-6P, *N*-acetylglucosamine-6-phosphate; ManNAc-6P, *N*-acetylmannosamine-6-phosphate; *Sa*NanE, NanE from *Staphylococcus aureus*.

**Figure 8:**
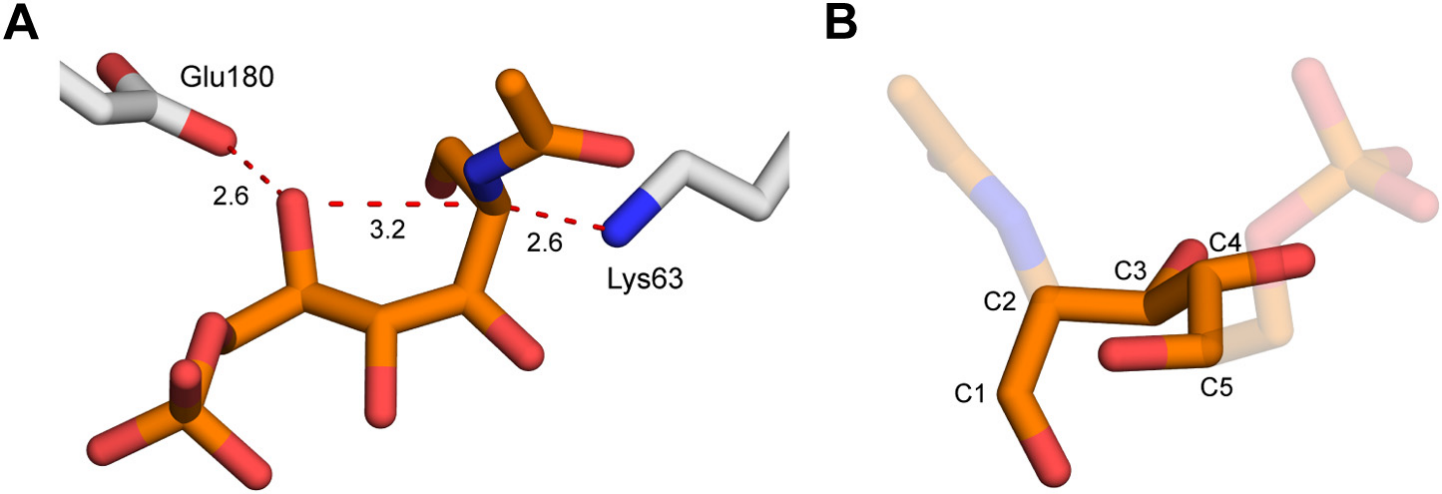
**The catalytic pair is positioned for catalysis.***A*, Lys63 and Glu180 are well positioned for deprotonation and substrate-assisted reprotonation. Distances between atoms are measured in Å. *B*, the substrate is arranged in a pseudochair conformation in the active site from C1 to C5 that positions the C5 hydroxyl for intramolecular proton transfer.

Based on our structural and kinetic data, we propose that NanE performs its epimerization reaction using a novel proton displacement mechanism mediated by the substrate (Fig. 7). In this proton displacement mechanism, Lys63 deprotonates the activated C2 position of ManNAc-6P to form an enolate intermediate that is stabilized by Arg40, Gln11, and Lys63. Once formed, the substrate hydroxyl at the C5 position, which is activated by Glu180, mediates reprotonation of the opposite face of the enolate from an ideal position to form GlcNAc-6P (Fig. 8*A*). The active site binds the substrate in a pseudochair conformation (Fig. 8*B*), which places the C5 hydroxyl in a position that would be ideal for hydrogen displacement to the C2 carbon, both in distance (3.2 Å) and angle (100°) (Fig. 8*A*).

In conclusion, we present a novel mechanism of epimerization by NanE involving substrate-assisted proton displacement and compared this to the previously proposed mechanism, which involves single-residue deprotonation–reprotonation. The substrate-assisted mechanism does not require residue translation or substrate rotation and is a new mechanism for carbohydrate epimerization. Collectively, our data and mechanistic interpretation may be useful in the development of inhibitors of this enzyme or in enzyme engineering to produce biocatalysts capable of changing the stereochemistry of molecules that are not amenable to synthetic methods.

## Experimental procedures

### Materials

ManNAc-6P derivatives, 5-deoxy-ManNAc-6P and 5-methyl-ManNAc-6P, were synthesized as reported (40).

### Cloning and mutagenesis

Constructs containing wildtype *Sa*NanE-encoding, *Sa*NagA-encoding, and *Sa*NagB-encoding genes from *S. aureus* USA300 were generated previously (9, 23). Substituted variants of *nanE* (Gln11Ala, Gln11Ser, Arg40Ala, Lys63Ala, Lys63Glu, Asp124Ala, Asp124Gln, Glu180Ala, and Arg208Ala) were produced commercially and supplied in a pET30a(+) expression plasmid (GenScript).

### Protein expression and purification

All constructs were expressed and purified as described previously for *Sa*NanE, *Sa*NagA, and *Sa*NagB (9, 23). Following size-exclusion chromatography, SDS-PAGE analysis showed that the final purity of each protein was at least 95% as judged by eye. To confirm protein identity, the mass of each purified protein was analyzed using a maXis 3G quadrupole time-of-flight mass spectrometer (Bruker Daltonics) equipped with an electrospray ionization source.

### X-ray crystallography

Crystallization studies were performed using protein concentrated to 10 to 20 mg/ml in 20 mM Tris (pH 8.0). Initial crystallization trials were either performed inhouse or at the Collaborative Crystallization Centre (C3) node of the Commonwealth Scientific and Industrial Research Organization (Melbourne, Australia). Apocrystals of wildtype *Sa*NanE and *Sa*NanE-Glu180Ala were successfully grown under the E1 conditions of the JCSG+ screen (1 M trisodium citrate, 0.1 M sodium cacodylate, and pH 6.5) at 8 or 20 °C using the sitting-drop vapor-diffusion method, with droplets consisting of equal volumes (150 or 200 nl) of protein solution and reservoir solution (23). Crystals were soaked in a cryoprotectant consisting of 7.5% (v/v) ethylene glycol, 7.5% (v/v) glycerol, and 85% (v/v) reservoir solution and flash frozen in liquid nitrogen. To obtain ligand-bound wildtype structures, 20 mM ManNAc-6P and 10 mM ManNAc-6P derivatives were cocrystallized with the protein under the same crystallization conditions.

The data were integrated and scaled using XDS (42) and AIMLESS (43, 44). Molecular replacement was performed using Phaser (45) in CCP4 (46). The monomer of PDB entry 1Y0E was used as the search model for the apo structure of *Sa*NanE, which was then used as the search model for all other structures within this study. Structure refinement was performed using REFMAC (47, 48) and phenix.refine (34). Iterative improvement of the map and the model was performed using alternate cycles of refinement and residue-by-residue analysis in COOT (34, 47, 48, 49). Coordinate and restraint files for 5-deoxy-ManNAc-6P were generated using phenix.elbow (50). Data collection and refinement statistics are presented in Table 1.

### Analytical ultracentrifugation

Sedimentation velocity experiments were conducted in an XL-I Analytical Ultracentrifuge (Beckman Coulter) using 0.5 mg/ml *Sa*NanE in 20 mM Tris (pH 8.0) and 150 mM NaCl. For these experiments, 400 μl of reference solution and 380 μl of sample were loaded into 12-mm double-sector cells with quartz or sapphire windows, and then mounted in an An-60 Ti rotor. Initial scans were performed at 3000 rpm to determine the optimal radial settings and wavelength. Data were collected at 285 nm, 50,000 rpm, and 20 °C. Radial absorbance data were collected at a single wavelength without averaging, using a 0.003-cm step size for a total of 80 to 120 scans. Solvent density, solvent viscosity, and an estimate of the partial specific volume for *Sa*NanE were computed using SEDNTERP (51). Data were fitted to a continuous size distribution model and a continuous mass distribution model using SEDFIT (52).

### SAXS

SAXS data were collected on the SAXS/WAXS beamline, equipped with a Pilatus 1M detector (170 × 170 mm; effective pixel size, 172 × 172 μm) at the Australian Synchrotron. The X-ray wavelength was 1.0332 Å. A sample-detector distance of 1600 mm was used, providing a *q* range of 0.006 to 0.4 Å^−1^. A 50-μl aliquot of sample at approximately 10 mg/ml was subjected to on-line size-exclusion chromatography on a Superdex 200 5/150 GL column (GE Healthcare) equilibrated with 20 mM Tris (pH 8.0) and 150 mM NaCl before entering the capillary for data collection.

### Spectrophotometric activity assay

An assay coupling the downstream enzymes of the sialic acid degradation pathway, NagA and NagB, with PGI and G6PD was developed (Fig. 5) (38). The pathway sequentially converts GlcNAc-6P into GlcN-6P, fructose-6-phosphate, and G6P. G6PD then converts G6P into 6PG, concomitantly reducing NADP^+^ to NADPH, which absorbs at 340 nm. The assay was extended from the coupled assay described for NagB (53).

To follow the coupled reaction, absorbance was measured at 340 nm as a function of time using a Cary 100 Bio UV/Vis spectrophotometer (Agilent Technologies). Initial rate measurements were performed in duplicate. Steady-state kinetic analysis of *Sa*NanE was performed at 25 °C. Reaction mixtures were equilibrated to 25 °C for 10 min prior to initiating the reaction by the addition of NanE. Standard 1-ml reaction mixtures contained 100 mM Tris (pH 8.0), 5 mM magnesium chloride, 1 mM NADP^+^, 50 μg of *Sa*NagA (produced inhouse), 100 μg of *Sa*NagB (produced inhouse), 50 μg of PGI (Sigma–Aldrich), 25 μg of G6PD (Sigma–Aldrich), and varying amounts of the substrate, ManNAc-6P (Carbosynth). The kinetic constants, *K*_*M*_, *V*_max_, and *k*_cat_, were determined using 0.16 to 5 mM ManNAc-6P for the wildtype and Glu180Ala enzymes and 1 to 5 mM ManNAc-6P for the other substituted enzymes. Initial rate data were fitted to the Michaelis–Menten model to determine the relevant kinetic constants (GraphPad Prism; GraphPad Software, Inc).

### Proton NMR activity assay

The activity of wildtype *Sa*NanE was measured as described previously (25, 38) at 25 °C with a Bruker Avance III 600 MHz instrument. The concentration of ManNAc-6P used in the assay varied from 0.16 to 5 mM. The final concentration of the enzyme was equal to that used in the spectrophotometric assay (0.25 μg/ml).

### Testing imine formation

To examine whether NanE uses an imine between Lys63 and the C1 aldehyde of ManNAc-6P, the mass of wildtype *Sa*NanE and that of the protein treated with its substrate (5 mM) plus sodium cyanoborohydride (50 mM) for 2 h were measured. Protein concentrations varied from 0.7 to 1.7 mg/ml for each sample. NAL and sialic acid were used as a comparison, as they are known to form an imine (11).

Separately, wildtype *Sa*NanE (1 mg/ml) was incubated in the presence of ManNAc-6P (5 mM) and sodium borohydride (100 mM) on ice for 30 min, after which the activity was measured using the spectrophotometric assay. NAL and sialic acid were used in a separate kinetic assay as a comparison (11).

### Plasmid availability

The plasmids for the coupling enzymes used in the spectrophotometric assay are available on request.

## Data availability

The atomic coordinates and structure factors (PDB IDs: 6VVA, 7MFS, 7MQT, and 7MFN) have been deposited in the PDB (http://wwpdb.org/).

## Supporting information

This article contains supporting information (54, 55).

## Conflict of interest

The authors declare that they have no conflicts of interest with the contents of this article.
