# Supplementary material for: N-acetylmannosamine-6-phosphate 2-epimerase uses a novel substrate-assisted mechanism to catalyze amino sugar epimerization
Source: J Biol Chem. 2021 Aug 24;297(4):101113. doi: 10.1016/j.jbc.2021.101113 (PMC8482478; doi:10.1016/j.jbc.2021.101113)
Supplement: Supplemental Figures S1–S7 and Tables S1, S2 [file mmc1.pdf]

Supporting information for

N-Acetylmannosamine-6-phosphate 2-epimerase uses a novel substrate-assisted mechanism to catalyze amino sugar epimerization

Michael J. Currie, Lavanyaa Manjunath, Christopher R. Horne, Phillip M. Rendle, Ramaswamy Subramanian, Rosmarie Friemann, Antony J. Fairbanks, Andrew C. Muscroft-Taylor, Rachel A. North & Renwick C. J. Dobson

Real-time spectrophotometric activity assay.

Three controls were performed to ensure that the coupled assay produced accurate results.

1) Specificity of the sequential assay coupling

The first was to test whether the coupling enzymes non-specifically catalyzed a reaction with the substrate of NanE, ManNAc-6P. Baseline absorbance of the assay mixture (100 mM Tris (pH 8.0), 5 mM magnesium chloride and 1 mM $NADP^+$) with the substrate, ManNAc-6P, was recorded in the absence of coupling enzymes. The coupling enzymes were then sequentially added to the mixture in reverse order (G6PD, PGI, NagB and NagA) to determine whether they were acting on the substrate. We observed activity in the absence of NanE when NagA was added (**Supp. Figure 5A, curve E**). This can likely be attributed to the presence of small amounts of product (GlcNAc-6P) in the commercially produced ManNAc-6P sample, which has a minimum purity of 95%. To remove residual GlcNAc-6P before initiating the reaction with NanE, we incubated the mixture for 10 min to ensure the contaminating product was converted to 6PG by the coupling enzymes.

2) Rate limitation

We confirmed that all of the coupling enzymes were in excess to ensure that the observed rate was not limited by any of the coupling enzymes. By doubling the concentration of each coupling enzyme and monitoring the reaction for any change in rate, we determined that 50 µg of NagA, 100 µg of NagB, 50 µg of PGI and 25 µg of G6PD per assay could be used as excess. At these concentrations, the rate of the reaction did not change when the concentration of each coupling enzyme was doubled (**Supp. Figure 5B**).

3) Linearity

The observed rate was determined to be proportional to NanE concentration between 0.25–2 µg/mL (**Supp. Figure 5C**). A final concentration of 0.25 µg/mL was selected for kinetic analysis of *Sa*NanE because it did not rapidly exceed the linear range of the spectrophotometer.

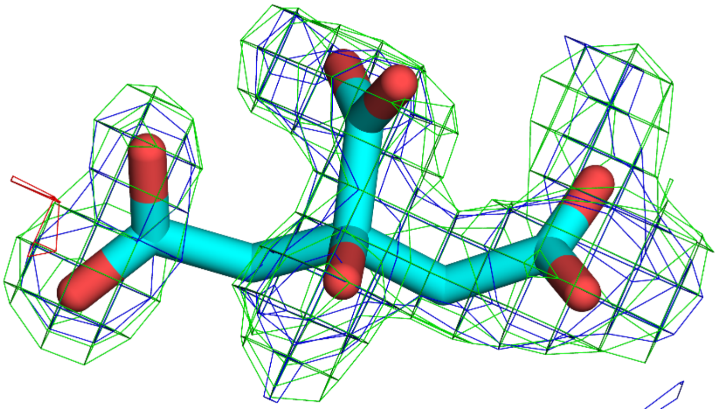

Figure S1 | An omit map of the active site density of apo *Sa*NanE (6VVA) (citrate overlaid) shows density consistent with citrate. The occupancy was modelled as 0.84 by phenix.refine. The $2F_o$ - F_c electron density map is contoured at 1.0 RMS (blue mesh) and the F_o - F_c omit electron density map is contoured at +3.0 σ (green mesh) and -3.0 σ (red mesh).

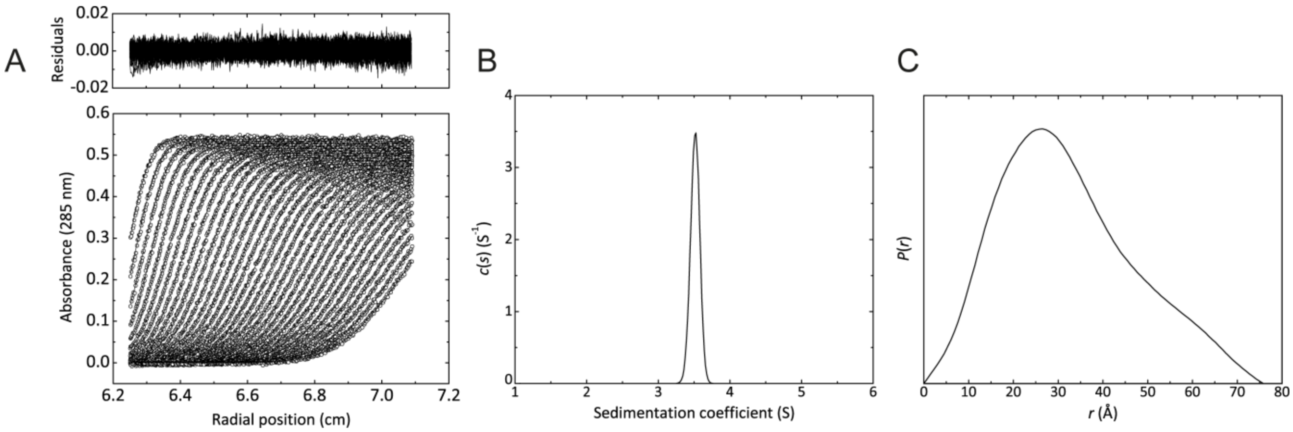

Figure S2 | A) Sedimentation velocity analysis of wild-type *Sa*NanE using analytical ultracentrifugation. Absorbance at 285 nm plotted as a function of radial position. The raw data are represented as open symbols (o) and overlaid with the non-linear least squares best fit to the *c*(*s*) model. The residuals for this fit are shown above. **B)** The sedimentation velocity data were fitted to the c(s) model and plotted as a function of sedimentation coefficient, giving a single component at 3.5 S and a frictional ratio (f/f_0) of 1.29. **C)** The real space distance distribution function *P*(*r*) plot showed that the maximum protein dimension is 75 Å.

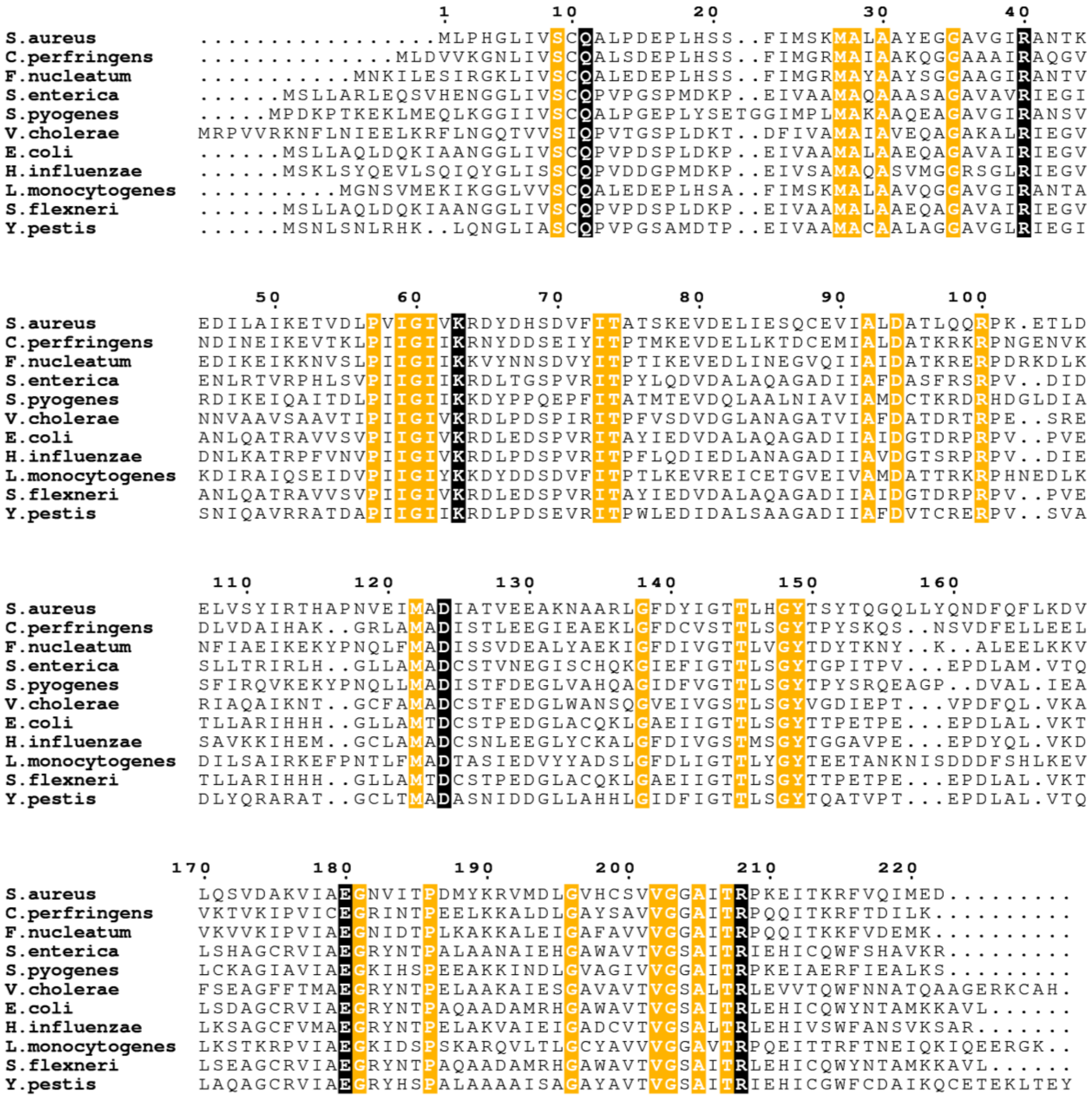

Figure S3 | Sequence alignment of *Sa*NanE to ten NanE sequences from other pathogenic bacteria. Conserved residues that were investigated for their involvement in catalysis are highlighted in black and other conserved residues are highlighted in orange. Species include both Gram-positive (*S. aureus*, *Listeria monocytogenes*, *C. perfringens*, and *S. pyogenes*) and Gram-negative bacteria (*Haemophilus influenzae*, *Shigella flexneri*, *S. enterica*, *F. nucleatum*, *V. cholerae*, *E. coli* and *Yersinia pestis*). Residues are numbered according to *S. aureus*. The alignment was generated using Clustal Omega (54) and modified using ESPript (55).

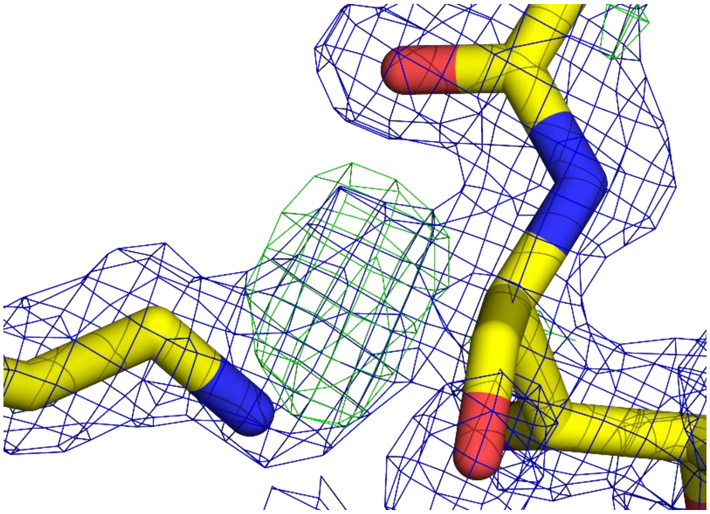

Figure S4 | Density between Lys63 and ManNAc-6P in the active site of monomer B of substrate/product bound *Sa*NanE (7MFS). The $2F_o$ - F_c electron density map is contoured at 1.0 RMS (blue mesh) and the F_o - F_c omit electron density map is contoured at +3.0 σ (green mesh).

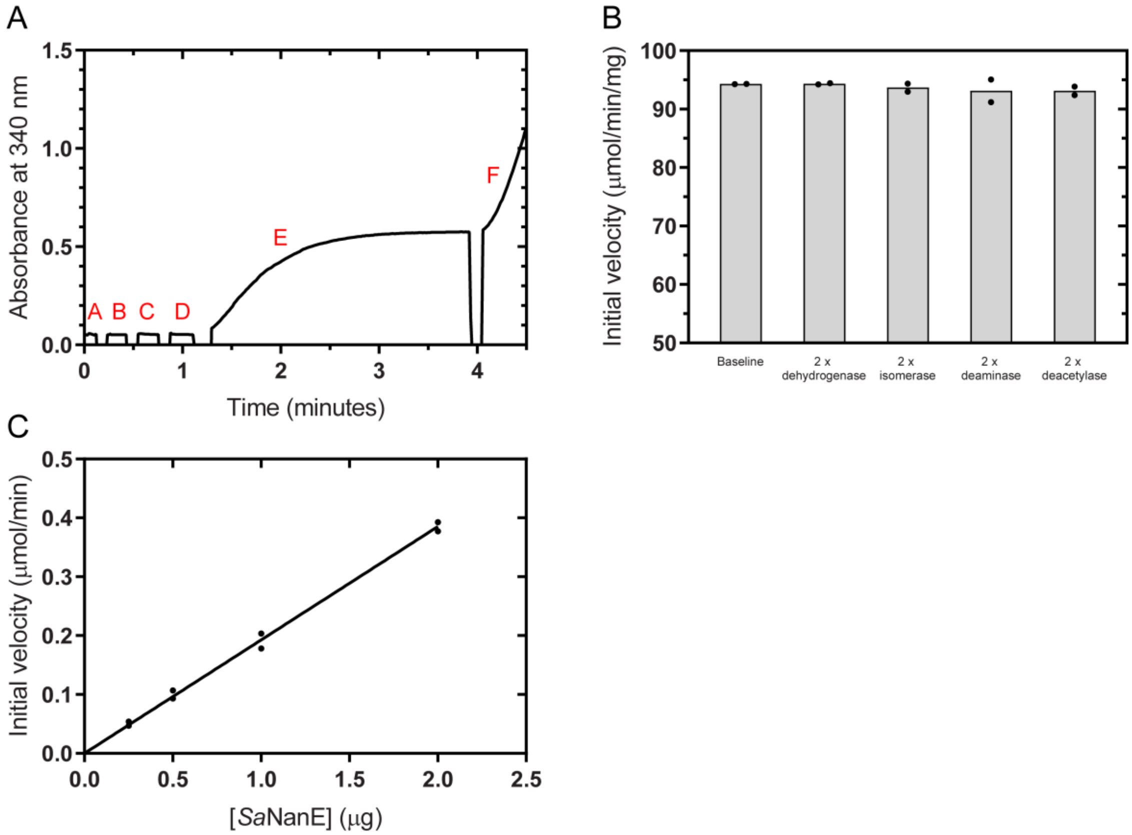

Figure S5 | Specificity, rate limitation and concentration linearity controls for the coupled assay. **A)** Absorbance at 340 nm as each enzyme was sequentially added in reverse order to the assay mixture. No increase in absorbance at 340 nm was observed in the absence of enzymes (A) or when G6PD, PGI and NagB were added to the assay (B, C and D, respectively). When NagA was added to the assay, an increase in absorbance at 340 nm, followed by a flattening of the curve, was observed (E). When NanE was added (F), the absorbance at 340 nm steeply increased to provide a curve, the data of which could be modelled by the Michaelis-Menten equation. **B)** Initial velocity of wild-type *Sa*NanE (baseline) with 50 µg of NagA, 100 µg of NagB, 50 µg of PGI and 25 µg of G6PD. The amount of each enzyme was sequentially doubled in reverse order. The mean is shown as a bar and the data is plotted as points. **C)** The initial velocity of wild-type *Sa*NanE as a function of enzyme concentration. Data were fitted to a linear model resulting in an R^2 value of 0.99.

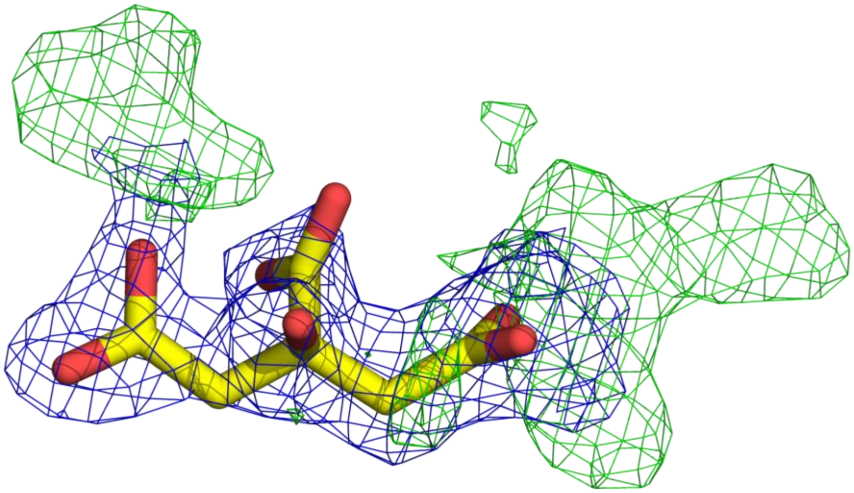

Figure S6 | Unambiguous density for the *N*-acetyl (left) and phosphate (right) groups of 5-deoxy-ManNAc-6P is present alongside density for citrate in an omit map of the active site of 7MQT. The $2F_o$ - F_c electron density map is contoured at 1.0 RMS (blue mesh) and the F_o - F_c omit electron density map is contoured at +3.0 σ (green mesh).

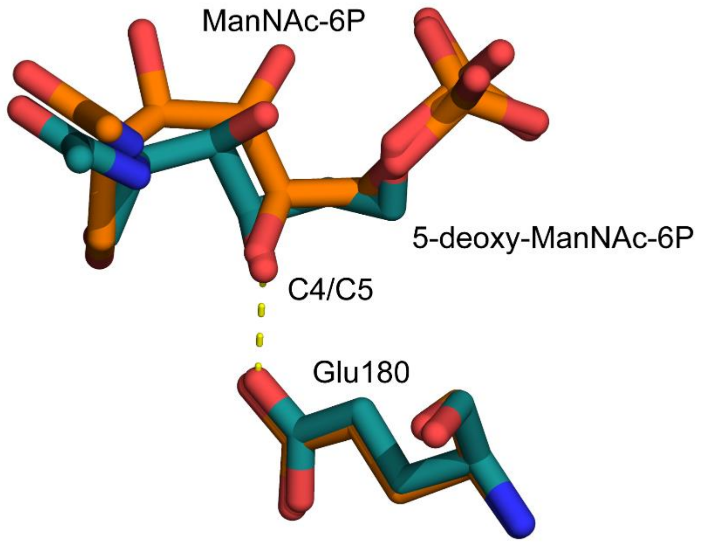

Figure S7 | 5-deoxy-ManNAc-6P (green ligand) binds in a different conformation to ManNAc-6P (orange ligand). The phosphate and ***N***-acetyl groups are similarly positioned. The C4 hydroxyl of the derivative binds in place of the C5 hydroxyl of ManNAc-6P, forming the close interaction with Glu180, which is in the same position between the structures. Citrate has been omitted for clarity.

Table S1 | Distances between Glu180 and the ligand C5 hydroxyl across all available structures. The density for the ligand in the lower resolution *Vc*NanE structures is unclear. The average distances are considered separately for these structures as the positions of the oxygen atoms are not well defined. (S) = substrate/ManNAc-6P, (P) = product/GlcNAc-6P.

	Chain in asymmetric unit				
	A	B	C	D	Average distance (Å)
**High resolution structures**					
*Sa*NanE (S) (1.51 Å)	2.53	2.61	-	-	2.57
*Sa*NanE (P) (1.51 Å)	2.64	2.63	-	-	2.64
*Sa*NanE average					2.60
*Cp*NanE (S) (1.45 Å)	2.61	2.59	-	-	2.60
*Cp*NanE (P) (1.90 Å)	2.53	2.66	2.29	2.41	2.47
*Cp*NanE average					2.54
SaNanE/CpNanE average					***2.57***
**Low resolution structures**					
*Vc*NanE (S) (2.66 Å)	2.89	3.00	-	-	2.95
*Vc*NanE (P) (2.66 Å)	3.10	3.13	-	-	3.12
*Vc*NanE average					3.03

Table S2 | Melting temperature of the wild-type and substituted *Sa*NanE enzymes, and the coupling enzymes *Sa*NagA and *Sa*NagB. Differential scanning fluorimetry was conducted at 1 mg/mL in 20 mM Tris, pH 8.0.

Enzyme	Melting temperature (°C)
Wild-type *Sa*NanE	40.0
*Sa*NanE- Lys63Ala	43.0
*Sa*NanE-Lys63Glu	40.7 ± 0.3
*Sa*NanE-Glu180Ala	47.8 ± 0.2
*Sa*NanE-Arg40Ala	41.3 ± 0.3
*Sa*NanE-Asp124Ala	44.0
*Sa*NanE-Asp124Gln	38.7 ± 0.3
*Sa*NanE-Gln11Ala	40.0
*Sa*NanE-Gln11Ser	39.3 ± 0.3
*Sa*NanE-Arg208Ala	42.0
*Sa*NagA	47.0
*Sa*NagB	43.0
